# Epidemiology of injuries in racket sports: a cross-sectional study of specific injuries within one olympic cycle

**DOI:** 10.1186/s13102-025-01150-1

**Published:** 2025-04-25

**Authors:** Christophe Lambert, Marc Banerjee, Ramona Ritzmann, Daniel Lachmann, Bernd Wolfarth, Casper Grim, Sven Shafizadeh, Markus Geßlein, Nicholas Peters, Noémie Reinert

**Affiliations:** 1 Department of Trauma and Orthopaedic Surgery, University of Witten/Herdecke, Cologne Merheim Medical Centre, Erich-Ollenhauer-Weg 7, 50354 Cologne, Köln Germany; 2 Department of Biomechanics, University of Freiburg, Freiburg, Germany; 3 Department of Orthopedics and Traumatology, University Hospital Basel, University of Basel, Basel, Switzerland; 4 Department of Sports Sciences, Division of Sports Medicine, Humboldt University of Berlin, Berlin, Germany; 5 Department of Trauma Surgery and Sports Traumatology, Sana Dreifaltigkeits-Krankenhaus Cologne, Cologne, Germany; 6 Department of Orthopedics and Traumatology, Paracelsus Private Medical University Nuremberg, Nuremberg, Germany; 7 Department of Molecular, Cellular and Developmental Biology, University of Colorado Boulder, Boulder, USA; 8 Atos Mediapark Klinik Köln, Cologne, Germany; 9 Department of Orthopedic, Trauma and Hand Surgery, Klinikum Osnabrück, Osnabrück, Germany

**Keywords:** Olympic, Elite, Athletes, Extremities, Injuries, Muscle, Cartilage, Ankle, Shoulder, Down time, Performance reduction

## Abstract

**Objective:**

Despite the growing interest in racket sports, injury prevalence, circumstances and severity as a function of gender, performance level and return to play have not been investigated to date. The aim was therefore to evaluate the occurrence of sport-specific injuries from a quantitative and qualitative perspective in the Olympic disciplines Tennis, Badminton and Table Tennis.

**Methods:**

Injury characteristics were recorded using a three-part questionnaire with 139 items, which was distributed through an online link. Racket sport-specific injuries and frequencies were assessed according to gender and performance level in one Olympic cycle (from the Olympic Games in London in 2012 until Rio de Janeiro in 2016). The Injury severity was recorded by time loss and performance reduction.

**Results:**

A total of 390 (55%) athletes have suffered a serious injury. There were more injuries during training than competition. 78% of the three most common injuries in the various racket sports involved the lower extremities. The longest injury time loss was seen in Tennis for an unspecified injury of the shoulder (16±12 weeks), in Badminton for the foot-ligament injury (13±14 weeks) and the meniscus injury of the knee in table tennis (13±10 weeks). The injuries to the knee accounted for the highest number of athletes with a reduced level of performance (Badminton: Knee - unspecified injury: reduced level=64%; Table tennis: Knee - meniscus injury: reduced level=54%)

**Conclusion:**

Although recognized as one entity of racket sports with some similar trends in terms of injuries, there are some relevant differences in injury type, circumstances and consequences, which should be considered in future sport-specific injury prevention strategies.

## Introduction

Racket sports like Tennis, Badminton and Table Tennis are competitive Olympic disciplines and gained increasing popularity since the last decade. Despite growing interest in racket sport, injury prevalence, injury circumstances and severity with reference to gender, level of performance and return to play have not been epidemiologically investigated, yet. Surprisingly for racket sports, in which motor skills and visual perception is executed with an emphasis on the upper body part, injuries are predominantly located in the lower extremity [1]. Acute accidents occur in the ankle joint, followed by injuries of the upper extremity which tend to be more of a chronic nature [2–4], as the lateral epicondylitis or known as “tennis elbow” or chronic shoulder instabilities and arthorisis [5]. Further, there are rapid multidirectional acceleration and deceleration movements seen in racket sports, making some anatomic regions, as the knee, more prone to injuries [6]

As with many other sports, the literature presents conflicting data regarding training-related versus competition-related injuries, often due to missing or inconclusive injury data or difficult assessment of overuse injuries [7].. Beside overlapping injury pattern with other non-contact sports, there are some unique considerations to racket sports, such as the impact of the different court surfaces played on [8]. Despite a shared preponderance of musculotendinous injuries [9] and the biomechanical and kinetic commonalities, there are sport-specific movement characteristics such as repetitive rapid jumps and lunges with an excessive load on lower extremity seen in Badminton [10]. Further long-term associations take place in the context of the injured body region and the affected structure, which are associated with consequences such as downtime and delayed regain of performance or permanently reduced performance.

In professional tennis, injuries to the knee and shoulder seem to be the most severe considering time loss from competition [11], often requiring a surgical treatment with the inability to return to previous performance level [12]. Although considered as low risk sports [9] table tennis, badminton and tennis were listed among the sports with the longest downtime rates after injury in the London summer Olympic Games 2012 [7]. Return-to-play is mainly determined by injury severity and level of play with some injuries, mostly acute ones, resulting in withdrawal from competition [8].

The aim of this study is to examine the prevalence and consequences of severe injuries among Racket sports disciplines in competitive and recreational athletes with respect to time loss from sport and reduction in sporting performance across genders, level of performance and affected body region. Long term data of 5 year were collected. The results of this study could provide a better understanding for injuries unique to racket sports athletes as further tailored prevention programs are needed.

## Material and methods

### Design of the epidemiological study

A retrospective survey was designed to document self-reported injury prevalence. The target period was an Olympic cycle from the London 2012 Summer Olympics to Rio de Janeiro 2016. An injury was defined as a training or competition break of more than 3 weeks, in accordance with the definition of Olsen et al. [13]. All injuries were based on the athletes'outputs and were consistently documented in writing; patient documentation was not included.

### Ethical approval

The authors were guided by the ethical principles of the “Declaration of Helsinki on Medical Research Involving Human Subjects”. The ethical application for disclosure was submitted to the Ethics Committee of Witten/Herdecke University and subsequently approved (Nr. 154/2017). At the beginning of the study, a written declaration of consent was obtained from all athletes. The athletes were informed about the anonymous use of the data for scientific purposes and the aggregated data.

### Survey

Injury characteristics were recorded using a three-part questionnaire with 139 items [14, 15]. The German Olympic Sports Confederation and the affiliated international sports federations published the questionnaire on their social-media accounts and sent it by email to their members to encourage athletes to participate. The survey consisted of the following three section:

1. The first section contained questions about the athletes'individual performance levels, which were divided into five performance categories: international elite, international, national, regional or recreational, indicating the highest level at which the athletes had competed in their career [15].
2. The second section contained questions about the athletes'injuries. Athletes were asked to indicate only serious injuries that resulted in more than 3 weeks of lost time [16]. For positive responses, the athlete indicated the type of injury, the anatomical region affected and the structure damaged. For each injury, age (years) at the time of occurrence, duration of downtime (weeks) and level of persistently performance after return to sport were assessed [15].
3. The third section contained data on anthropometry, age at completion of the survey and gender.

### Differentiation by anatomical topography and affected body structure

To assign the injuries to specific body regions, they were categorized into seven subgroups by body region into back, front torso, head, upper extremity excluding shoulder (ws), shoulder, knee and lower extremity excluding knee (wk). Furthermore, the injuries were categorized according to the damaged structure as follows: Torn ligament, cartilage damage, tendon injury and bone fracture.

### Study population

A total of 10606 athletes participated in the study. The exclusion criterion was an incomplete questionnaire. The results of 7809 complete surveys were included in the analyzes. For this study we only analyzed athletes taking part in racket sports, which accounted in 9,1% (*n=*709) of the analyzed surveys.

An age < 18 years Athletes classified as competing as international or national elite were pooled into the ‘competitive sports’ category. Athletes classified as competing in regional level or not taking part in competition in their sports were grouped into the ‘recreational sports’ category.

There was no sample size analysis used to draw a conclusive statement about the required number of enrolled subjects. But we used a maximum of subjects we could recruit with the expectation that the more numbers we have the more valuable the outcome would be.

### Statistical analysis

Only specific injuries with more than three events were analyzed in terms of lost time and reduction in athletic performance. Only injuries or injury-related questions with ten or more responses were included in the analysis.

We used SPSS 25.0 (SPSS, Inc, Chicago, Illinois, USA) for the statistical analyses encompassing descriptive statistics and significance tests. To compare injury prevalence throughout the Olympic cycle (2012–2016), we used χ^2^] tests. To analyze the top eight injury diagnoses and the discipline-specific top three diagnoses, between disciplines, gender, competitive or recreational athletes, and whether they occurred during training or competition, as well as the performance after the injury, we calculated χ^2^ tests. Since the duration of sporting time loss in weeks is a non-normal, interval scales variable, we computed Mann-Whitney-U tests (gender and competitive level) or Kruskal-Wallis tests for a comparison of the duration of sporting time loss between the top eight diagnoses.

*P*-values < 0.05 were statistically significant.

## Results

A total of 390 (55%) athletes reported to have suffered a serious injury in the time period of the Olympic Games in London in 2012 until the Olympic Games in Rio de Janeiro in 2016. In Badminton (62%; *n=*203) athletes reported significantly more injuries than in Tennis (50%; *n=*91) and Table tennis (47%; *n=*96) (*p>*0.001).

### Top 10 ranking of injuries and top three ranking of injuries differentiated by sports

Among all racket sports and genders the ligament injuries of the upper ankle (*n=*66) and the foot (*n=*65) showed the highest prevalence. In the top 10 ranking eight of ten injuries occurred in the lower extremities. For the upper extremities, the unspecified shoulder injury (*n=*57) and the muscle injury of the elbow (*n=*20) where the only two injuries in the top 10 ranked injuries.

Regarding the top three injuries in the different racket sports discipline seven (78%) of the nine injuries occurred in the lower limb region. Ligament injuries to the foot or the upper ankle joint were ranked in the top three ranking equally for Badminton, Tennis and Table tennis. (Badminton: Upper ankle joint- ligament injury= 14%; *n=*45; Foot- ligament injury= 13%; *n=*44) (Tennis: Foot – ligament injury= 8%; *n=*12) (Table tennis: Upper ankle joint- ligament injury= 7%; *n=*11) (Table 1).

**Table 1:** Top three injuries in the differentsports with reference to injury rate among all athletes and injuries

	**Most common injuries**	**N (Injuries)**	**Specific injury rate in relation to all injuries** **Injuries/Olympiade/Injuries**	**Specific injury rate in relation to athletes** **Injuries/Olympiade/Athlete**	**% (of all athletes with specific injury)**
**Badminton**	1) Upper ankle joint- ligament injury	45	14	0,027	14
	2) Foot - ligament injury	44	13	0,027	13
	3) Knee - unspecified injury	24	7	0,015	7
**Tennis**	1) Fibula - muscle injury	19	13	0,021	10
	2) Shoulder - unspecified injury	14	9	0,015	8
	3) Foot - ligament injury	12	8	0,013	7
**Table tennis**	1) Shoulder - unspecified injury	19	13	0,019	9
	2) Knee - mensicus injury	14	9	0,014	7
	3) Upper ankle joint- ligament injury	11	7	0,011	5

### Prevalence of the top three injuries during training and competition

**Fig. 1:**
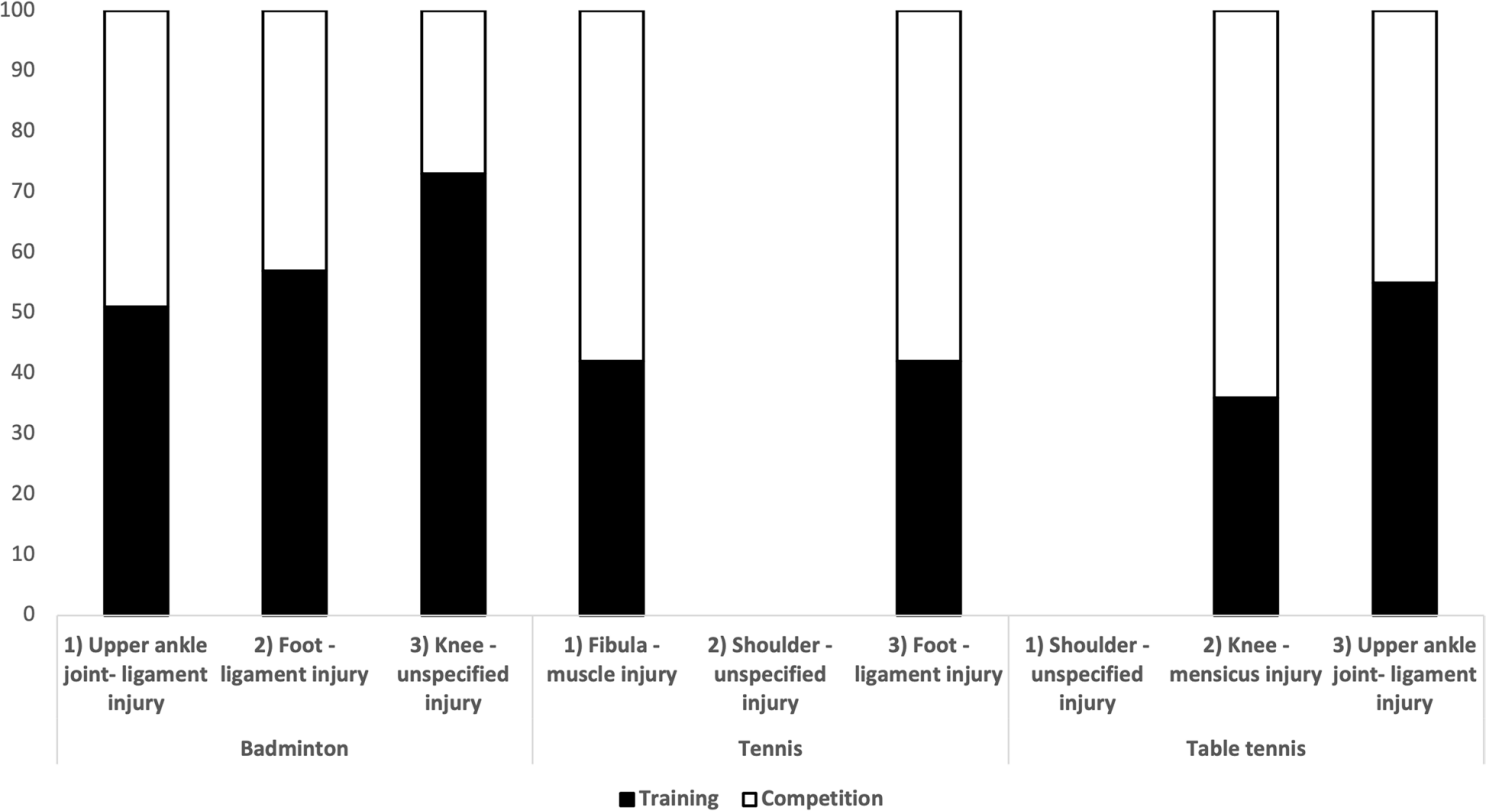
Injury prevalence illustrated as a distribution regarding training and competition distinguished by racket sports in Tennis, Badminton and Table Tennis. Note that the onset of unspecific shoulder injuries could not be attributed to training and competition as the chronic injury type is not associated to s specific origin

The occurrence of the top three injuries during training and competition varied between the sports. In Badminton a higher prevalence in training of the top three injuries was reported (Badminton: training injuries: Upper ankle joint- ligament injury= 51%; *n=*23; Foot- ligament injury= 57%; *n=*25; Knee- unspecified injury= 73%; *n=*8). In Tennis and Table tennis the athletes reported a higher prevalence of injuries during competition (Tennis: competition injuries: Fibula- muscle injury= 58%; *n=*11; Foot- ligament injury= 58%; *n=*7) (Table tennis competition injuries: Knee- meniscus injury= 64%; *n=*9). There were no significant differences regarding the genders and the competitive level of athletes (Fig. 1).

### Duration of sporting time loss

**Fig. 2:**
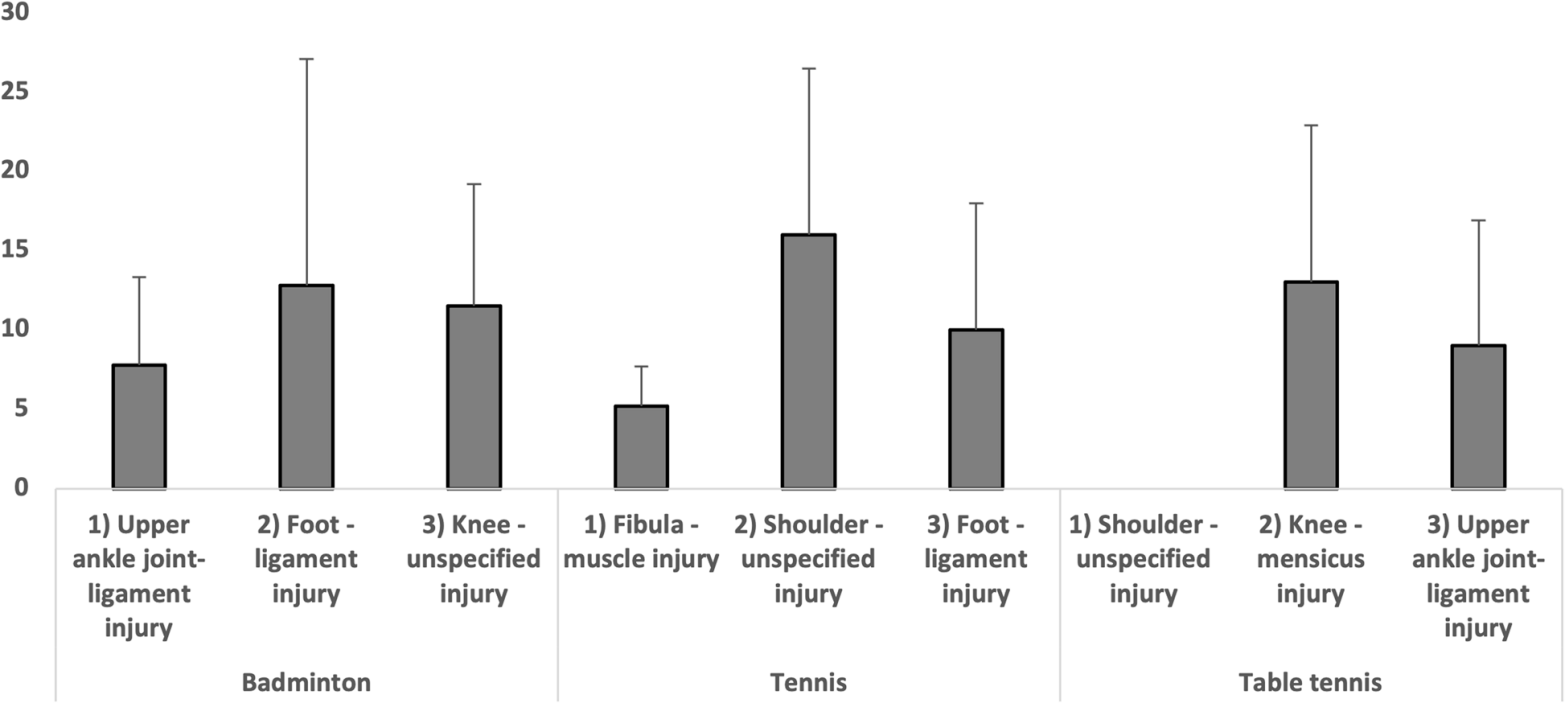
Downtime after injury for the different racket sports

All injuries in the top three ranking showed a time loss of less than 15 weeks. The longest time loss after injury was documented in Tennis for an unspecified injury of the shoulder (16 weeks). The foot-ligament injury accounted for the longest time loss in Badminton (13 weeks) and the meniscus injury of the knee in Table tennis (13 weeks). The injury with the shortest time loss in sporting activity was the fibula- muscle injury in tennis (5 weeks). Comparing the duration of time loss, there were no significant difference regarding the genders and the competitive level of athletes (Fig. 2).

### Reduction of performance

**Fig. 3:**
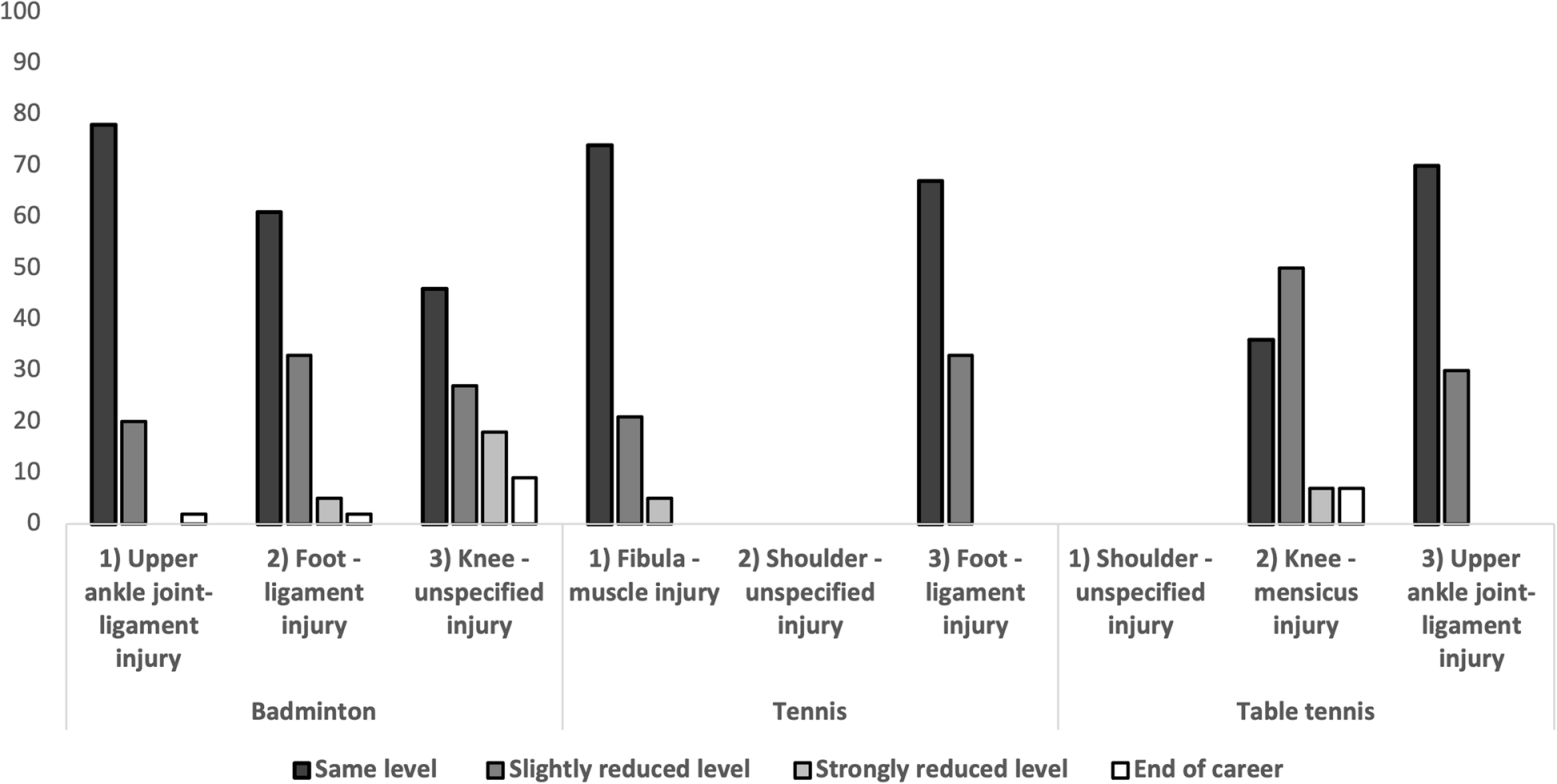
Rates of athletes that experienced a significant and persistent loss of performance after the injury in racket sports

The injuries to the knee accounted for the highest number of athletes with a reduced level of performance (Badminton: Knee- unspecified injury: reduced level= 64%; Table tennis: Knee- meniscus injury: reduced level= 54%). Three injuries in top ranking showed numbers of over 70% with the same level of performance after injury (Badminton: Upper ankle joint- ligament injury= 78%; Tennis: Fibula- muscle injury= 74%; Table tennis: Upper ankle joint- ligament injury= 70%). There was no significant difference regarding the genders and the competitive level of athletes comparing the reduction of performance after injury (Fig. 3).

## Discussion

This study provides an important insight into lost time, injury severity and the impact of gender and level of competition, differentiated by racket sport, with the following key findings.

### Top ranking of injuries differentiated by sports

The data in literature is unanimous: most injuries in Racket Sports occur to the lower limb [4, 9, 11, 14], followed by the upper extremity and the trunk [2, 15]. Injuries to the upper ankle joint, as ankle sprains, are considered to be the most common acute injuries [2, 15, 16] which is consistent with our findings. Others studies In a study analyzing injuries in Hong Kong elite badminton players injuries to the shoulder and knee were the most common types [17]. Kondric found a preponderance of shoulder injuries among top Slovenian racket sports athletes, with the highest incidence in table tennis, where the vulnerability of the shoulder complex is due to short and abrupt multiplanar movements [18]. With the highest incidence in tennis, Kondric found the ankle and foot to be the second most injured anatomic part in racket sports, which can be explained by a poor local muscle support and stabilization highlighting the need for good footwear [18]. The lower limb biomechanics and the different pattern of footwork generate high plantar and joint forces also in table tennis [19] leading to ankle sprains [20] as well as chronic conditions as plantar fasciitis [5], the jumper’s knee or knee osteoarthritis [6]. Also in badminton players repetitive loading to the patellar tendon, as seen in lunges, is associated with high intra-tendinous flow and the onset of the jumper’s knee [21].

### Duration of sporting time loss

Although underrepresented, injuries to the shoulder result in the longest time loss rates. In the literature, injuries to the upper extremities are often referred to as overuse injuries [2, 4, 15]. In a study of Young et al., only 25% of professional tennis players came back to their pre-injury level with a mean return to play time of 7 months after a shoulder surgery [12]. Kaldau et al. reported a lasting limitation in 50% of elite badminton players by significant injury with a mean time loss of 90 days [22]. In others sports acute injuries caused higher times loss compared to overuse injuries [23, 24]. Thereby, scientific evidence underpins a direct link between match and training load and number of injuries as well as the consequent time loss [11]

### Reduction of performance

Knee injuries in racket sports are inevitable and are responsible for a significant drop in performance. Inadequate warm-up is recognized as a primary injury factor, and a large number of athletes require surgical intervention in the form of knee arthroscopy, with meniscal injuries being the most common cause [25]. Powell et al. described a comparable performance achievement as prior to the injury although knee injuries are most often documented as severe injuries [11]. We have observed a remarkable reduction in performance of more than 50% in table tennis players with knee meniscus injuries, which is a cause for concern.

### Prevalence of the top three injuries during training and competition

There is evidence that more injuries occur in racket sports during competition than during training. In contrast, our study shows a higher prevalence of training injuries in badminton. This is in line with the findings of Phomsoupha et al. [26], mentioning the highest injury frequency at the end of training which could be related to muscle fatigue as a result of repetitive jumps and multidirectional stop-and-go maneuvers. Herbaut and Delannoy found an increased risk of ankle sprains due to fatigue in badminton players [27].

However, contradictory findings exist an refer to more competition-related injuries in their study [28]. Considering the US Open Tennis Championships between 1994 and 2009, Sell et al. reported more competition related injuries. However, it is mentioned by the authors that injuries which did not occur during match play were not generally collected by the medical staff and included in the study [16]. McCurdie et al. analyzed the injury data of the Wimbledon tournaments from 2003 - 2012 pointing out an onset of 61% of injuries prior to the arrival to the Championships shedding light on the importance of injury prevention during a professional tennis season [14]. Other authors described higher training related injuries for tennis [15] or badminton [17]. Table tennis presents the lowest injury rates, compared to tennis and badminton [29].

#### Limitations

As the data in the following study is based on retrospective documentation and self-reporting by athletes, there may be an over- or under-reporting of injuries, which should be considered as one of the main limitations. In addition, discrepancies or misstatements in injury rates may be due to the fact that injuries are reported at a selected point in time and not consecutively among the entire season [11]. In our study, no direct distinction was made between acute and overuse injuries, which may be a reasonable distinction based on literature.

### Prospect

Assessment of time loss and performance reduction following injury in racket sports shows discipline-specific injury patterns, with shoulder, knee and foot injuries causing the highest loss of performance and calcium. This study points to the importance of training injuries and emphasizes the need for future research on this topic to adapt training management and improve medical care and rehabilitation in all sports.

## Data Availability

The datasets generated and/or analysed during the current study are not publicly available due to not finished analysis on this data, which are not published until this moment. But are available from the corresponding author on reasonable request.
